# Tethered Bilayer Lipid Membrane Platform for Screening Triton X-100 Detergent Replacements by Electrochemical Impedance Spectroscopy

**DOI:** 10.3390/nano13050874

**Published:** 2023-02-26

**Authors:** Sue Woon Tan, Negin Gooran, Hye Min Lim, Bo Kyeong Yoon, Joshua A. Jackman

**Affiliations:** 1 School of Chemical Engineering and Translational Nanobioscience Research Center, Sungkyunkwan University, Suwon 16419, Republic of Korea; 2 School of Healthcare and Biomedical Engineering, Chonnam National University, Yeosu 59626, Republic of Korea; 3 Interdisciplinary Program of Biomedical Engineering, Chonnam National University, Yeosu 59626, Republic of Korea

**Keywords:** Triton X-100, detergent, lipid bilayer, tethered bilayer lipid membrane, critical micelle concentration, electrochemical impedance spectroscopy

## Abstract

In light of regulatory considerations, there are ongoing efforts to identify Triton X-100 (TX-100) detergent alternatives for use in the biological manufacturing industry to mitigate membrane-enveloped pathogen contamination. Until now, the efficacy of antimicrobial detergent candidates to replace TX-100 has been tested regarding pathogen inhibition in endpoint biological assays or probing lipid membrane disruption in real-time biophysical testing platforms. The latter approach has proven especially useful to test compound potency and mechanism of action, however, existing analytical approaches have been limited to studying indirect effects of lipid membrane disruption such as membrane morphological changes. A direct readout of lipid membrane disruption by TX-100 detergent alternatives would be more practical to obtain biologically relevant information to guide compound discovery and optimization. Herein, we report the use of electrochemical impedance spectroscopy (EIS) to investigate how TX-100 and selected replacement candidates—Simulsol SL 11W (Simulsol) and cetyltrimethyl ammonium bromide (CTAB)—affect the ionic permeability of tethered bilayer lipid membrane (tBLM) platforms. The EIS results revealed that all three detergents exhibited dose-dependent effects mainly above their respective critical micelle concentration (CMC) values while displaying distinct membrane-disruptive behaviors. TX-100 caused irreversible membrane disruption leading to complete solubilization, whereas Simulsol caused reversible membrane disruption and CTAB induced irreversible, partial membrane defect formation. These findings establish that the EIS technique is useful for screening the membrane-disruptive behaviors of TX-100 detergent alternatives with multiplex formatting possibilities, rapid response, and quantitative readouts relevant to antimicrobial functions.

## 1. Introduction

Controlling microbial contamination is a major challenge in the biological manufacturing industry [1,2,3]. Impurities and contamination not only degrade product quality but also pose consumer safety risks and interfere with production facility operations [4]. Furthermore, identifying and investigating contaminants in biological production streams imposes high costs, is time-intensive, and requires extensive resources. To ensure product safety and quality, various purification methods such as acidic pH conditions, chromatography, detergent treatment, and nanofiltration have been implemented in manufacturing process streams to eliminate pathogenic contaminants such as viruses, bacteria and fungi [5,6,7]. Detergent treatment is considered the most effective method to inactivate membrane-enveloped viruses and typically involves the use of membrane-disruptive agents [8]. Within this scope, the Triton X-100 (TX-100) detergent has been widely used as a viral inactivation agent due to its ability to disrupt the membrane envelope surrounding virus particles and other pathogens [9,10]. However, a degradation product of TX-100, 4-tert-octylphenol, is a known endocrine disruptor [11], prompting discussion about public health safety and possible bioremediation strategies [12,13]. These issues have also led to restricted usage of TX-100 in the European Union and the European Chemicals Agency’s listing of TX-100 as a contaminant of emerging concern has prompted ongoing efforts to identify TX-100 detergent replacements [14]. 

From a biophysical perspective, an ideal detergent replacement for TX-100 would have a comparable ability to inactivate membrane-enveloped viruses, which is principally related to disrupting and solubilizing phospholipid membranes [15,16]. To date, the effects of TX-100 treatment on membrane permeability have been mainly investigated using endpoint biological assays focused on measuring pathogen inhibition efficacy [17,18,19] or, in more limited cases, with solution-based fluorescence spectroscopy and microscopy experiments that typically involve the release of encapsulated dye [8,20,21]. In addition to measuring inactivation efficiency, there remains an outstanding need to characterize the mechanism of action of TX-100 detergent replacements to validate them before entering manufacturing lines [22,23]. To gain insight into the biophysical interactions between detergents and lipid membranes with high speed and efficiency, label-free biosensing techniques have been developed to track corresponding changes in the physical, chemical, and electrical properties of cell membrane-mimicking phospholipid membranes. Among the different options, the supported lipid bilayer (SLB) platform—a two-dimensional lipid bilayer coating on silica-based surfaces—has been widely used with various surface-sensitive measurement techniques [24,25]. For example, using the quartz crystal microbalance-dissipation (QCM-D) technique, Gooran et al. recently compared the membrane-disruptive interactions of TX-100 and a proposed replacement, the nonionic surfactant, Simulsol 11-W (Simulsol), using a supported lipid bilayer (SLB) platform, and discovered that Simulsol only caused reversible membrane morphological changes whereas TX-100 caused irreversible and complete membrane solubilization [26]. While prior biological studies had shown that TX-100 and Simulsol reduce the infectivity of certain viruses to a similar level [18], this biophysical measurement approach unequivocally showed that the two detergents have distinct membrane-interaction profiles and highlighted the importance of incorporating biophysical experiments into TX-100 replacement screening and mechanistic evaluation frameworks.

Another important point of the biophysical measurement readout involves considering how relevant is the tracked interaction process to the intended antimicrobial function? While the QCM-D technique measures membrane morphological changes due to detergent interactions, this readout does not directly reflect changes in membrane permeability, which is more closely related to membrane-targeting antimicrobial functions [27]. As such, it would be desirable to expand the scope of biophysical testing possibilities to include a direct readout of membrane permeability changes and one excellent sensing option involves electrochemical impedance spectroscopy (EIS) to track changes in the ionic permeability of a tethered bilayer lipid membrane (tBLM) platform on a gold electrode surface [28,29]. The tBLM consists of a phospholipid bilayer coating on top of a sparsely tethered, self-assembled monolayer made up of different-length molecules (i.e., long tethers and short spacers) to create an ionic reservoir that facilitates ion transfer across the lipid bilayer [30]. Using the EIS technique, ion transfer can be measured by tracking the time-resolved conductance (G_m_) signal and, since the lipid bilayer serves as an insulator, the time-resolved capacitance (C_m_) signal can also be measured and provide information about membrane thickness [31,32]. While the tBLM platform with EIS monitoring has been mainly used to study membrane-active peptides [33] until now, it is also capable of detecting anionic and nonionic surfactant interactions [29] and thus has excellent potential for testing TX-100 detergent replacement candidates.

**Figure 1:**
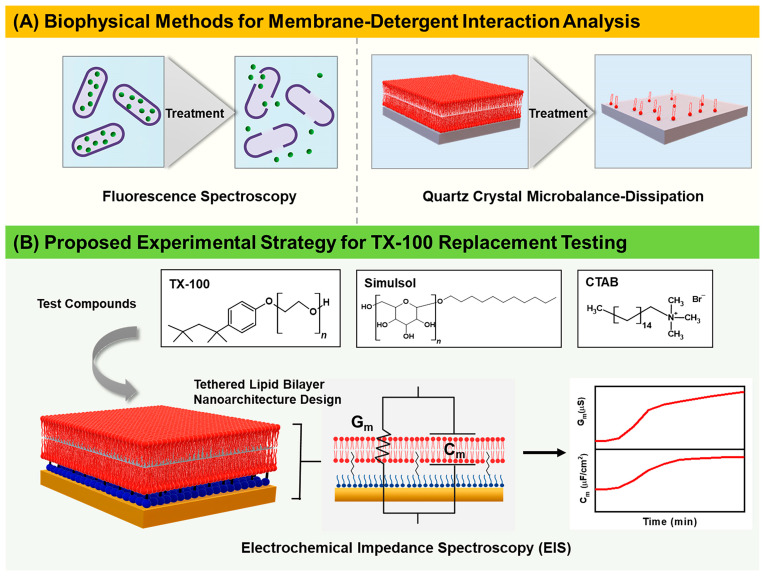
Overview of experimental strategy to evaluate membrane-disruptive properties of TX-100 detergent replacement candidates. (**A**) Commonly used biophysical methods for membrane-detergent interaction analysis require labeling and/or only provide indirect readouts of ionic permeability changes across lipid bilayers. (**B**) Proposed experimental strategy for TX-100 replacement testing based on electrochemical impedance spectroscopy (EIS). The molecular structures of the test compounds, TX-100, Simulsol, and CTAB are presented and defined bulk concentrations of each compound were individually added to the tethered bilayer lipid membrane (tBLM) platform. Time-resolved conductance (G_m_) and capacitance (C_m_) signals were monitored to evaluate how each compound disrupts the tBLM platform.

Towards this goal, herein, we employed the EIS technique to comparatively investigate how TX-100 and two replacement candidates—Simulsol and cetyltrimethyl ammonium bromide (CTAB)—disrupt tBLM platforms and ascertain the extent to which different membrane-disruptive behaviors can be distinguished (Figure 1). TX-100 and Simulsol are both nonionic surfactants, and Simulsol in particular consists of an undecyl-alcohol hydrophobic chain and glucose moiety that has a low environmental impact [34]. On the other hand, CTAB is a cationic surfactant commonly used in pharmaceutical and food sectors, including in the downstream purification process, for membrane disruption-related cell component extraction and antimicrobial purposes [35,36,37]. By utilizing the EIS technique and considering the critical micelle concentration (CMC) value of each compound, it was identified that TX-100 caused irreversible, complete membrane solubilization, that Simulsol induced reversible membrane disruption, and that CTAB treatment resulted in irreversible, albeit incomplete, membrane defect formation. With the growing number of TX-100 detergent replacements being explored, these proof-of-principle results establish that the EIS testing approach is suitable for screening TX-100 detergent replacement candidates with various charge properties and reaffirm that TX-100 and Simulsol have distinct membrane-interaction profiles while also establishing a biophysical framework to classify suitable types of membrane disruption. Compared to other biophysical testing options, we may also add that the EIS technique enabled label-free, direct tracking of ionic permeability changes, whereas other commonly used techniques require either labeling and/or only provide indirect readouts. As such, the EIS measurement capabilities can be extended to screen a wider range of TX-100 replacement candidates in the future and to potentially probe finer distinctions in membrane-disruptive properties between candidates.

## 2. Materials and Methods

### 2.1. Materials

Triton X-100 (TX-100), hexadecyl-trimethylammonium bromide (CTAB), and 1-pyrenecarboxaldehyde (1-pyCHO) were supplied by Sigma-Aldrich (St. Louis, MO, USA). Simulsol SL 11W (Simulsol) was provided by Seppic Inc. (Fairfield, NJ). A 10× phosphate-buffer saline (PBS) solution (Gibco, Carlsbad, CA, USA) was diluted to 1× with Milli-Q-treated deionized water (MilliporeSigma, Burlington, MA, USA), and the solution pH was then adjusted to pH 7.2. The PBS buffer solution was used to prepare all samples.

### 2.2. Detergent Sample Preparation

Initial sample preparation was done at the highest test concentration of each compound. TX-100 and Simulsol samples were prepared by dissolving the desired volumes in PBS buffer. The CTAB sample was made by dissolving the appropriate mass in PBS buffer. The samples were vortexed and then heated for 30 min at 70 °C to aid dissolution. After cooling, serial dilution was carried out to prepare the desired test concentrations immediately before experiments. 

### 2.3. Critical Micelle Concentration (CMC) Measurements

The critical micelle concentration (CMC) of CTAB was determined using a SpectraMax iD5 microplate reader (Molecular Devices, San Jose, CA). Wavelength-shift spectroscopy measurements were conducted by using 1-pyCHO as the fluorescent probe, as previously described [38]. Based on the fluorescence properties of 1-pyCHO, the excitation wavelength of 366 nm and emission wavelength range from 410 to 600 nm were set. 1-pyCHO was dissolved in methanol to produce a 5 mM methanolic stock solution. Then, aliquots of the stock solution were transferred to glass vials and dried at room temperature to allow methanol evaporation for at least 30 min. Next, the dried 1-pyCHO was hydrated with PBS solution containing varying CTAB concentrations, while the final 1-pyCHO concentration was fixed at 0.1 μM for experiments. The average of a minimum of four technical replicates was used to determine the maximum-intensity emission wavelength for each compound concentration. The CMC values of TX-100 and Simulsol were also obtained using the same protocol and were previously reported [26].

### 2.4. Electrochemical Impedance Spectroscopy (EIS)

Gold-patterned glass slide pre-functionalized with a mixed monolayer composed of tether (benzyldisulphide polyethylene glycol C20-phytanyl) and spacer (hydroxyl-terminated benzyldisulphide tetraethylene glycol) molecules in 1:9 molar ratio was supplied by SDx Tethered Membranes (Sydney, Australia) for tethered bilayer lipid membrane (tBLM) formation. The slide surface was cleaned with ethanol before being mounted onto a tethaPlate cartridge (SDx Tethered Membranes) with six flow cells. The solvent-exchange procedure was carried out on the monolayer-coated surface in each flow cell to fabricate the tBLM. Briefly, 8 μL of 3 mM ethanolic lipid solution consisting of 70 mol% C_20_ diphytanyl-diether-phosphatidylcholine lipid and 30 mol% C_20_ glycerol diphytanyl ether lipid (SDx Tethered Membranes) was injected, and then rinsing with 3 × 100 μL aliquots of PBS buffer was performed according to the manufacturer’s protocol. To characterize tBLM formation, the tethaPlate cartridge was inserted into the tethaPod instrument (SDx Tethered Membranes). ElS measurements were conducted to obtain the conductance (G_m_) and capacitance (C_m_) signals of the tBLM platforms and were run by using tethaQuick software (SDx Tethered Membranes). Under zero bias potential, the EIS was operated with 25 mV alternating current (AC) excitation at frequencies ranging from 0.1 Hz to 2000 Hz. tBLM formation was validated by G_m_ values of ~0.6 μS and C_m_ values of 1.0–1.4 μF/cm^2^, which served as the experimental baseline values. All measurements were repeated at least thrice, and data were analyzed using the OriginPro (OriginLab, Northampton, MA, USA) software package. In applicable cases, statistical analysis was performed using GraphPad Prism8 software (GraphPad Software, San Diego, CA, USA). A one-tailed, unpaired t-test was used for comparison between two groups and one-way analysis of variance (ANOVA) using Tukey’s multiple comparison test was used for comparison between multiple groups. *P*  <  0.05, *P*  <  0.01, *P*  <  0.001, and *P*  <  0.0001 indicate the statistical significance levels. 

## 3. Results

### 3.1. Real-Time EIS Measurements

We conducted EIS measurements to investigate how the TX-100 detergent and replacement candidates disrupted the tBLM platform in terms of affecting ionic permeability across the lipid bilayer. The EIS technique is well-suited for tracking changes in the conductance (G_m_) and specific capacitance (C_m_) signals of the tBLM platform when different bulk concentrations of detergent were added into the measurement chamber. The ionic space between the gold electrode surface and tBLM platform permits ions to cross the membrane and the ion flow specifics are highly sensitive to the membrane properties. While a fully intact membrane has high electrical sealing, defect formation within the membrane can cause greater ion flow across the lipid bilayer due to increased membrane permeability and lead to a larger G_m_ signal [28]. Changes in the C_m_ signals can also arise from membrane thinning events, for example. Furthermore, the effects of detergents on tBLM properties may be assessed by using a Bode plot that compares phase minima shifts before and after treatment as well as after subsequent buffer washing to remove bulk compound from the measurement chamber. The G_m_ and C_m_ signals corresponding to tBLM formation in PBS served as the measurement baseline signals prior to adding appropriate concentrations of the detergent compound in PBS to the measurement chamber. After the compound addition, there was a 30-min incubation period followed by buffer washing.

### 3.2. TX-100

**Figure 2:**
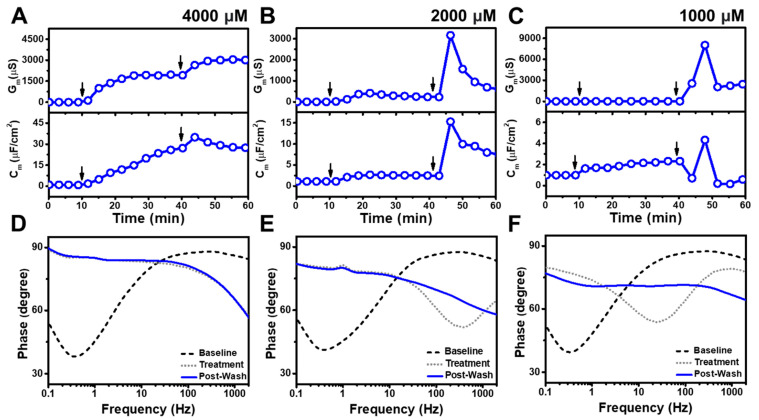
EIS measurements for a tBLM treated with TX-100 above its CMC value. Time-resolved conductance (G_m_) and specific capacitance (C_m_) signals following the addition of (**A**) 4000 μM, (**B**) 2000 μM, and (**C**) 1000 μM TX-100. Sequential arrows indicate compound addition and buffer washing. (**D**–**F**) Corresponding Bode plots showing phase minima shifts vs. frequency for the tBLM baseline (Baseline), after TX-100 addition (Treatment), and after buffer rinsing (Post-Wash).

Figure 2 presents the EIS results for TX-100 addition to the tBLM platform at different bulk concentrations above its CMC value of 300 μM. When 4000 μM TX-100 was added, it induced increases in both the G_m_ and C_m_ signals to around 1960 μS and 23 μF/cm^2^ respectively (Figure 2A). The signals increased appreciably after buffer washing, reaching values around 3000 μS and 25 μF/cm^2^ respectively. Upon 2000 μM TX-100 addition, the G_m_ and C_m_ signals rose to around 230 μS and 3 μF/cm^2^ respectively (Figure 2B). The G_m_ signal further increased after buffer rinsing and peaked at 3160 μS before progressively declining to 690 μS. A C_m_ signal of around 8 μF/cm^2^ was also recorded with a similar response profile. For 1000 μM TX-100 addition, the G_m_ and C_m_ signals increased to around 20 μS and 2.3 μF/cm^2^ respectively (Figure 2C). Upon buffer washing, the G_m_ signal appreciably rose to around 8000 μS before stabilizing at around 2400 μS, whereas the C_m_ signal remained steady at approximately 0.5 μF/cm^2^. Interestingly, these data support that TX-100 causes extensive disruption in all cases, however, the initial G_m_ shift upon detergent treatment demonstrated high TX-100 concentration dependency, whereby the G_m_ signal upon detergent treatment was roughly 10-times higher for the 4000 μM vs. 2000 μM cases and also ~10-times higher for the 2000 μM vs. the 1000 μM cases. For the 4000 μM TX-100 case, the Bode plot also showed appreciable phase minima shifts to higher phase and frequency after detergent treatment. It remained unchanged after buffer rinsing, which is a typical response for membrane solubilization (Figure 2D). On the other hand, according to the Bode plots for the 2000 and 1000 μM TX-100 cases, the phase minima shifted to larger frequency and phase following detergent treatment, indicating partial solubilization while even further shifting to a higher phase after buffer washing, which indicated that membrane solubilization was more extensive upon buffer washing in those cases (Figure 2E,F).

**Figure 3:**
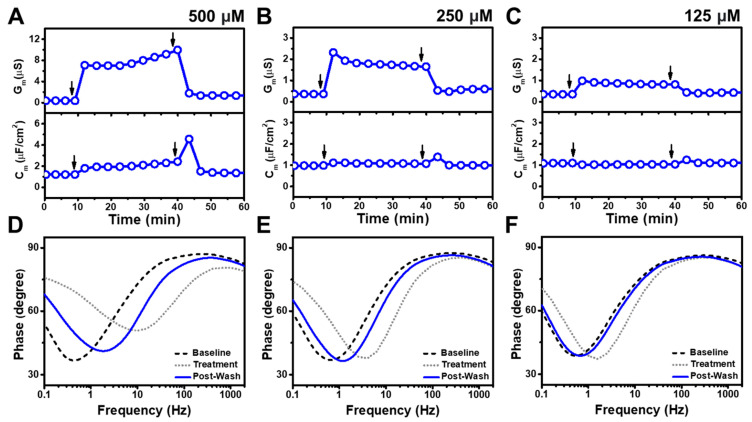
EIS measurements for a tBLM treated with TX-100 around and below its CMC value. Time-resolved conductance (G_m_) and specific capacitance (C_m_) signals following the addition of (**A**) 500 μM, (**B**) 250 μM, and (**C**) 125 μM TX-100. Sequential arrows indicate compound addition and buffer washing. (**D**–**F**) Corresponding Bode plots showing phase minima shifts vs. frequency for the tBLM baseline (Baseline), after TX-100 addition (Treatment), and after buffer rinsing (Post-Wash).

We also tested lower TX-100 concentrations to identify the lowest concentration at which appreciable membrane disruption occurs (Figure 3). At 500 μM TX-100 concentration, the G_m_ and C_m_ signals increased to around 8.6 μS and 2 μF/cm^2^, respectively, after which the buffer rinsing step caused the signals to decrease to around 1.3 μS and 1 μF/cm^2^, respectively (Figure 3A). Below the CMC value of TX-100, 250 μM TX-100 addition led to only slight increases in the G_m_ and C_m_ signals to around 1.7 μS and 1 μF/cm^2^, respectively (Figure 3B). Following buffer rinsing, the signals decreased to around 0.6 μS and 1 μF/cm^2^. When 125 μM TX-100 was added, the measurement responses were even smaller and the G_m_ signal barely increased to around 0.8 μS before falling to 0.4 μS, similar to the baseline value prior to compound addition (Figure 3C). A negligible C_m_ signal shift was also observed. 

In contrast to the higher test concentrations, the Bode plot analysis showed that 500 μM TX-100 treatment caused irreversible lipid membrane disruption, but not extensive solubilization, as indicated by a phase minima shift to higher frequency and phase [29] (Figure 3D). After buffer washing, the frequency and phase values only partially returned to the baseline values. In addition, 250 and 125 μM TX-100 treatment caused minor phase minima shifts, but the phase minima returned to almost their initial states after buffer rinsing (Figure 3E,F). These findings are consistent with concentration-dependent TX-100 disruption above the CMC and minor, reversible interactions below CMC. More specifically, at 4000 μM TX-100 concentrations, there was extensive membrane solubilization upon treatment while 2000 and 1000 μM TX-100 concentrations treatment caused extensive membrane disruption upon treatment and further induced solubilization upon buffer washing. Conversely, 500 μM TX-100 treatment caused irreversible membrane disruption to a much lesser extent while 250 and 125 μM TX-100 treatment had only minor, largely reversible effects.

### 3.3. Simulsol

**Figure 4:**
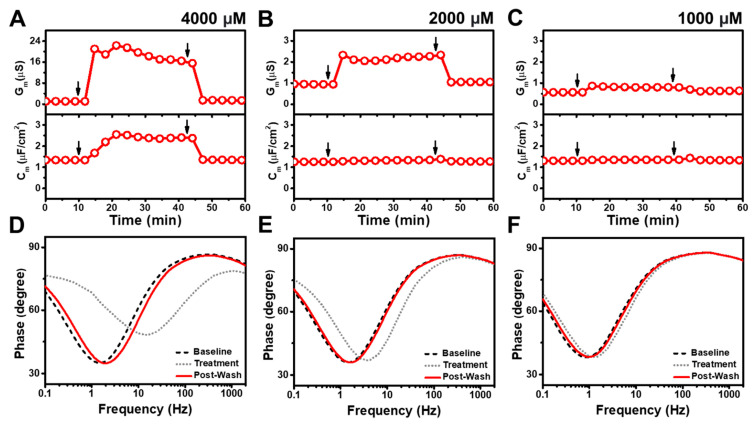
EIS measurements for a tBLM treated with Simulsol at different concentrations. Time-resolved conductance (G_m_) and specific capacitance (C_m_) signals following the addition of (**A**) 4000 μM, (**B**) 2000 μM, and (**C**) 1000 μM Simulsol. Sequential arrows indicate compound addition and buffer washing. (**D**–**F**) Corresponding Bode plots showing phase minima shifts vs. frequency for the tBLM baseline (Baseline), after Simulsol addition (Treatment), and after buffer rinsing (Post-Wash). Note that 4000 μM Simulsol is above CMC and 2000 and 1000 μM Simulsol are below CMC.

Figure 4 shows the time-resolved G_m_ and C_m_ shifts caused by Simulsol addition, at concentrations above and below its CMC value of 2300 μM, to the tBLM platform. A 4000 μM Simulsol addition led to an increase in the G_m_ signal of around 16 μS and a modest increase in the C_m_ signal by around 2 μF/cm^2^ (Figure 4A). After buffer washing, the G_m_ and C_m_ signals decreased to around 1.4 μS and 1 μF/cm^2^, respectively, indicating largely reversible membrane disruption. Below its CMC value, 2000 μM Simulsol only slightly increased the G_m_ signal to around 2.3 μS before returning to its baseline of about 1 μS due to buffer washing (Figure 4B). There was no change in the C_m_ signal. When 1000 μM Simulsol was added, there was a modest rise in the G_m_ signal to around 0.8 μS, that returned to around 0.6 μS after buffer washing (Figure 4C). A negligible C_m_ signal shift was again observed. At even lower Simulsol concentrations, the G_m_ and C_m_ shifts due to compound treatment were negligible (Figure S1). 

Bode plot analysis demonstrated that 4000 μM Simulsol treatment caused reversible membrane disruption due to the phase minima shifting to higher frequency and phase after compound addition but nearly returning to baseline values after buffer washing [29,32] (Figure 4D). For the 2000 μM and 1000 μM Simulsol cases, the phase minima shifted slightly to higher frequencies, but eventually returned to the initial baseline values (Figure 4E,F). Overall, Simulsol demonstrated reversible membrane disruption to an appreciable extent only above its CMC and was largely inactive below its CMC.

### 3.4. CTAB

While Simulsol has been suggested to replace TX-100, CTAB is a cationic, quaternary ammonium surfactant that also displays membrane-disruptive antimicrobial properties. Depending on the solution conditions, its reported CMC value can range from around 60 μM to 900 μM [37,38,39,40]. In PBS, electrostatic interactions between the cationic hexadecyl-trimethylammonium ion and anionic phosphate ion contribute to CTAB micellization, whereby charge neutralization at the micellar surface reduces monomer repulsion to facilitate micelle formation at a relatively low CMC [41]. Accordingly, we measured the CMC of CTAB in PBS to be 50 μM, which is consistent with the aforementioned mechanism (Figure S2), and selected appropriate test concentrations of CTAB above and below the CMC for subsequent EIS measurements.

**Figure 5:**
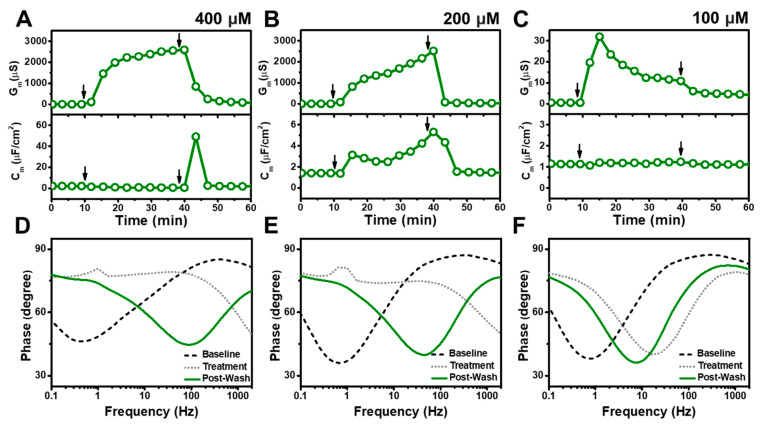
EIS measurements for a tBLM treated with CTAB above its CMC value. Time-resolved conductance (G_m_) and specific capacitance (C_m_) signals following the addition of (**A**) 400 μM, (**B**) 200 μM, and (**C**) 100 μM CTAB. Sequential arrows indicate compound addition and buffer washing. (**D**–**F**) Corresponding Bode plots showing phase minima shifts vs. frequency for the tBLM baseline (Baseline), after CTAB addition (Treatment), and after buffer rinsing (Post-Wash).

Figure 5 presents the time-resolved G_m_ and C_m_ shifts for CTAB addition to the tBLM platform at high concentrations above its CMC. When 400 μM CTAB was added to the tBLM, the G_m_ and C_m_ signals increased to approximately 2560 μS and 0.7 μF/cm^2^, respectively (Figure 5A). After buffer washing, the corresponding signals decreased to roughly 109 μS and remained around 2 μF/cm^2^. Upon 200 μM CTAB addition, the G_m_ and C_m_ signals also increased to about 2510 μS and 5.3 μF/cm^2^, respectively, while the signals significantly decreased to 30 μS and 1.5 μF/cm^2^ after buffer washing (Figure 5B). These large responses due to 400 and 200 μM CTAB treatment indicate extensive, solubilization-like membrane disruption during the interaction process while the post-treatment G_m_ and C_m_ values indicate smaller, albeit still appreciable levels of irreversible disruption. On the other hand, upon 100 μM CTAB addition, the G_m_ signal increased up to 32 μS and gradually decreased to around 11 μS, with only a modest rise in the C_m_ signal of around 1.2 μF/cm^2^ (Figure 5C). After buffer washing, the G_m_ and C_m_ signals dropped to around 4.8 μS and 1.1 μF/cm^2^, respectively, indicating irreversible membrane disruption. Further analysis of the Bode plots showed that 400 μM and 200 μM CTAB caused solubilization-like disruption upon treatment, as indicated by phase minima shifts to higher frequencies (Figure 5D,E). However, after buffer washing, the phase minima moderately returned to lower frequencies, supporting irreversible membrane disruption but not complete solubilization. Similar effects were also seen in the 100 μM CTAB case that points to irreversible membrane disruption, while the magnitude of the signal shifts was smaller compared to the highest CTAB concentration cases (Figure 5F).

**Figure 6:**
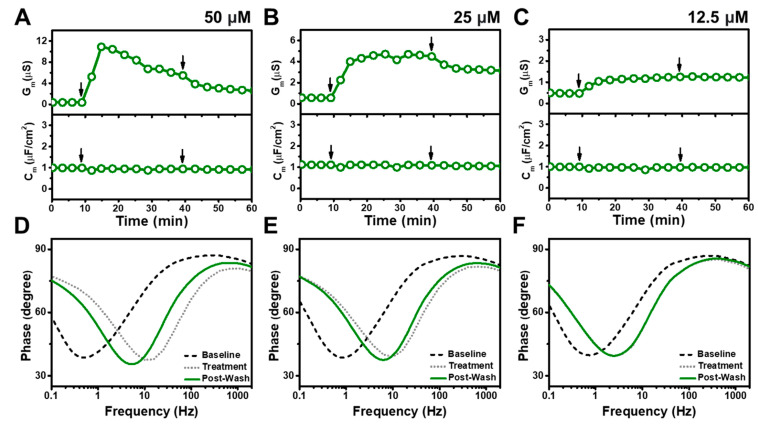
EIS measurements for a tBLM treated with CTAB at and below its CMC value. Time-resolved conductance (G_m_) and specific capacitance (C_m_) signals following the addition of (**A**) 50 μM, (**B**) 25 μM, and (**C**) 12.5 μM CTAB. Sequential arrows indicate compound addition and buffer washing. (**D**–**F**) Corresponding Bode plots showing phase minima shifts vs. frequency for the tBLM baseline (Baseline), after CTAB addition (Treatment), and after buffer rinsing (Post-Wash).

Figure 6 shows the time-resolved G_m_ and C_m_ shifts for CTAB addition to the tBLM platform at lower concentrations at and below its CMC. For 50 μM CTAB addition, the G_m_ signal quickly increased to reach a peak value of about 10 μS before gradually stabilizing at about 6.7 μS (Figure 6A). After buffer washing, the G_m_ signal decreased and remained steady at around 3 μS. The magnitude of the G_m_ measurement response became smaller at lower concentrations, and 25 μM CTAB addition led the G_m_ signal to increase slightly to around 4.7 μS (Figure 6B). After buffer washing, the G_m_ signal slightly decreased to around 3.2 μS. Likewise, upon 12.5 μM CTAB addition, the G_m_ signal had a minor and gradual increase to around 1.2 μS, where it remained stable even after buffer washing (Figure 6C). Overall, the magnitudes of the G_m_ shifts decreased at lower concentrations while there were negligible changes in the C_m_ signals for all tested CTAB concentrations below CMC. The corresponding Bode plots demonstrated that CTAB addition below CMC still caused irreversible membrane disruption although to a less extent than higher CTAB concentrations (Figure 6D–F). 

## 4. Discussion

**Figure 7:**
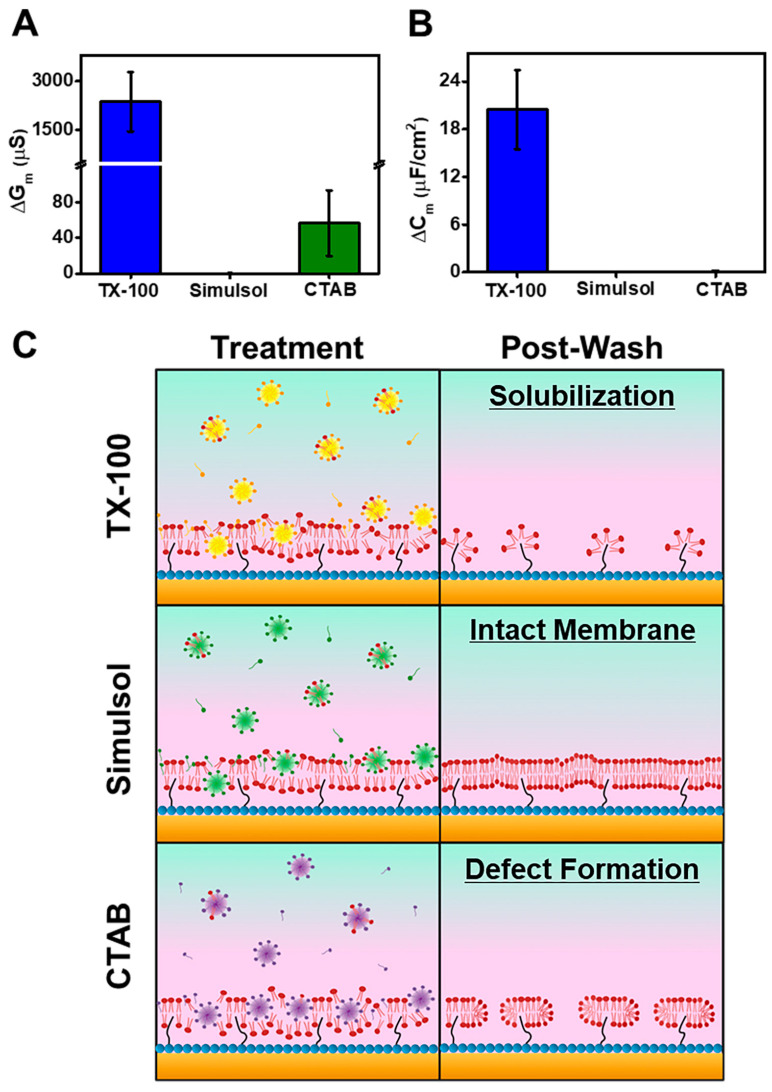
Experimental summary of the final (**A**) G_m_ and (**B**) C_m_ shifts for the tBLM platform after treatment with 4000 μM TX-100, 4000 μM Simulsol, or 400 μM CTAB followed by buffer washing (*n* = 3 independent experiments). In panel (**A**), the differences between TX-100 vs. Simulsol and TX-100 vs. CTAB were statistically significant (*P* < 0.01) by one-way ANOVA. In panel (**B**), the differences between TX-100 vs. Simulsol and TX-100 vs. CTAB were also statistically significant (*P* < 0.001). (**C**) Representative illustrations of the EIS results depicting the tBLM conditions after treatment with the highest test concentrations of TX-100, Simulsol, and CTAB (Treatment) and after buffer washing (Post-Wash).

A summary of key findings is presented in Figure 7 and describes quantitatively and illustratively how the EIS technique can characterize the membrane-disruptive properties of TX-100 and potential replacements. Figure 7A,B report the resulting G_m_ and C_m_ shifts after detergent treatment and subsequent buffer washing. At the highest test concentrations, treatment with 4000 μM TX-100 led to a G_m_ shift of 2372 ± 913 μS and a C_m_ shift of 20.4 ± 5 μF/cm^2^, which was followed by treatment with 400 μM CTAB that caused a G_m_ shift of 56.5 ± 36 μS and a C_m_ shift of 0.06 ± 0.18 μF/cm^2^. These values indicate that TX-100 and CTAB both caused irreversible membrane disruption. By contrast, the G_m_ and C_m_ shifts induced by 4000 μM Simulsol were only 0.07 ± 0.15 μS and 0.02 ± 0.05 μF/cm^2^, respectively, which indicate reversible membrane disruption only. Based on these EIS results, a schematic illustration of the different membrane-interaction behaviors for each compound is described in Figure 7C, and the corresponding mechanistic details and EIS measurement analysis are described below.

TX-100 induced the most pronounced membrane-disruptive activity by not only disturbing tBLM lipid packing but also, interestingly, causing more extensive membrane solubilization after buffer washing at TX-100 concentrations above CMC (≥1000 μM). The latter effect was signified by dramatically increased G_m_ and C_m_ shifts, the typical EIS response for membrane solubilization [28,29]. In line with previous QCM-D results that utilized SLB platforms, our EIS results demonstrate that TX-100 functions as a relatively slow-acting, solubilizing agent to disrupt the tBLM platform [15,26]. To rationalize this behavior, we may recall that TX-100 has curvophilic shape, i.e., positive spontaneous curvature, characteristic of detergents and can be attributed to the large conformational freedom of its hydrophilic moiety. As such, membrane insertion can lead to localized distortions of lipid bilayer packing that can cause the tBLM platform to curve outward [15]. Since the lipid bilayer was destabilized by this outwardly induced membrane curvature, the shear stress induced by the subsequent buffer washing step likely triggered the experimentally observed membrane solubilization.

On the other hand, the equivalent concentration of Simulsol also caused membrane disruption, through the original membrane properties were restored upon buffer rinsing with negligible G_m_ and C_m_ shifts recorded relative to the tBLM baseline. These findings agreed with the past QCM-D experiments [26] and support that TX-100 and Simulsol have distinct membrane-interaction profiles. Besides the relatively high CMC of Simulsol, the pyranose ring on the hydrophilic part of Simulsol may constrain its conformational freedom, impeding Simulsol from deeply penetrating the lipid bilayer. As such, it is likely that interacting Simulsol micelles remain near the lipid bilayer surface and hence are more easily removed upon buffer washing. This steric-type blocking effect also helps to explain why the membrane-disruptive effects of Simulsol were reversible, i.e., it could perturb the outer region of the lipid bilayer structure but could not deeply penetrate to cause membrane solubilization. 

In contrast to the nonionic properties of TX-100 and Simulsol, CTAB is a cationic detergent that also disrupted the tBLM packing properties. After buffer washing, relatively large G_m_ shifts remained, indicating irreversible membrane disruption. Even so, CTAB induced a minimal C_m_ shift so it is likely that not all of the membrane was removed, i.e., complete solubilization did not occur. Our findings are consistent with past research that demonstrated CTAB is largely ineffective at fully removing SLBs despite extensive disruption [42]. Contrary to how the well-known, anionic SDS works to fully solubilize the tBLM platform [29], the EIS data supports that CTAB is more suited to enhancing membrane permeability, consistent with its reported activity to facilitate molecular exchange across cell membranes [43]. 

Another noteworthy point concerns the concentration-dependent effect of the two tested nonionic detergents vs. the tested cationic detergent. The major membrane-disruptive effects of TX-100 and Simulsol mainly occurred only above their respective CMC values while TX-100 was more potent due to having a lower CMC. On the other hand, the cationic CTAB detergent still exhibited some degree of irreversible membrane disruption even below its CMC, which likely relates to electrostatic interactions between cationic CTAB molecules and the slightly negatively charged lipid bilayer surface. It should also be noted that the C_14_ hydrocarbon chain of CTAB contributed to a relatively low CMC, thus reflecting its high potency. A direct comparison of TX-100 and CTAB at lower bulk concentrations (400 µM and below) further showed that CTAB is more potent than TX-100 (i.e., exerts membrane-disruptive effects at a lower concentration) and CTAB caused appreciably larger changes in the G_m_ signal at 400 µM concentration as well (Figures S3 and S4).

## 5. Conclusions

In this study, we employed the EIS technique to comparatively evaluate the membrane-disruptive effects of TX-100, Simulsol, and CTAB detergents on a tBLM platform, within the broader context of developing a measurement approach to assess molecular candidates that might replace TX-100 as a membrane-disrupting antimicrobial mitigant in biological manufacturing processes. While other biophysical methods such as the QCM-D technique have been previously used to investigate membrane morphological changes caused by TX-100 and Simulsol, the measurement readouts provide only indirect information about the membrane interaction process itself. It would be more desirable to directly evaluate membrane permeabilization, i.e., changes in ionic permeability, associated with membrane-related antimicrobial functions. The EIS technique fills this gap while providing label-free, rapid, scalable, and quantitative readouts of membrane permeability changes and corresponding time scales. Such insights allowed us to discriminate the mechanisms of action of TX-100 and replacement candidates, Simulsol and CTAB, leading us to conclude that these two candidates have distinct potency levels and corresponding disruption mechanisms compared to TX-100. Regarding evaluating membrane-disruptive behaviors on the tBLM platform, the EIS technique is well positioned for screening future TX-100 replacement candidates and can be more broadly used to evaluate industrially relevant detergent-membrane interactions for discovery and quality control among various application possibilities.

Regarding future directions, we may also note a wide range of TX-100 replacement candidates are being discussed in the literature and that have mainly been studied using biological assays [17,18,44]. It would be opportune to connect the EIS measurement capabilities with testing these various compounds and establishing correlates between biophysical and biological readouts that build off the measurement validation covered in the present study. One important point in this direction also covers the tBLM composition. In this study, we used an archaebacteria lipid-based composition with particularly good electrical sealing properties for tBLM platform fabrication, though it would be useful to expand to using lipid compositions that are more representative of mammalian and viral membranes. Inspired by nanoarchitectonic design principles [45], the latter point could be particularly significant when attempting to draw correlations between the membrane-disruptive interactions detected in the EIS readouts and biological assay results such as drops in viral infectivity. Building confidence in the predictive power of the EIS measurement readouts would support translation from fundamental membrane biophysics studies to use as an industry tool enabling accurate and quick assessment of TX-100 replacement candidates.

## Data Availability

The data presented in this study are available upon reasonable request from the corresponding author.

## Supplementary Materials

The following supporting information can be downloaded at: https://www.mdpi.com/article/10.3390/nano13050874/s1, Figure S1: Time-resolved G_m_ and C_m_ shifts for low Simulsol concentrations; Figure S2: Critical micelle concentration (CMC) measurement of CTAB using wavelength-shift fluorescence spectroscopy; Figure S3: EIS measurements for a tBLM treated with TX-100 at equivalent concentrations to those in the CTAB experiments; Figure S4: Experimental summary of direct comparison between 400 μM TX-100 and CTAB treatment effects.
