# Supplementary material for: Tethered Bilayer Lipid Membrane Platform for Screening Triton X-100 Detergent Replacements by Electrochemical Impedance Spectroscopy
Source: Nanomaterials (Basel). 2023 Feb 26;13(5):874. doi: 10.3390/nano13050874 (PMC10005542; doi:10.3390/nano13050874)
Supplement: Supplementary file 1 [file nanomaterials-13-00874-s001.zip › nanomaterials-2230778-supplementary.pdf]

Tethered Bilayer Lipid Membrane Platform for Screening Triton X-100 Detergent Replacements by Electrochemical Impedance Spectroscopy

Sue Woon Tan [1], Negin Gooran [1], Hye Min Lim [2,3], Bo Kyeong Yoon [2,3,*] and Joshua A. Jackman [1,*]

1. School of Chemical Engineering and Translational Nanobioscience Research Center, Sungkyunkwan University, Suwon 16419, Korea; suewoon4695@gmail.com (S.W.T.); negingrn@gmail.com (N.G.)
2. School of Healthcare and Biomedical Engineering, Chonnam National University, Yeosu 59626, Korea; shlthgks@naver.com (H.M.L.)
3. Interdisciplinary Program of Biomedical Engineering, Chonnam National University, Yeosu 59626, Korea

* Correspondence: bkyoon@jnu.ac.kr (B.K.Y.); jjackman@skku.edu (J.A.J.)

Supporting Information

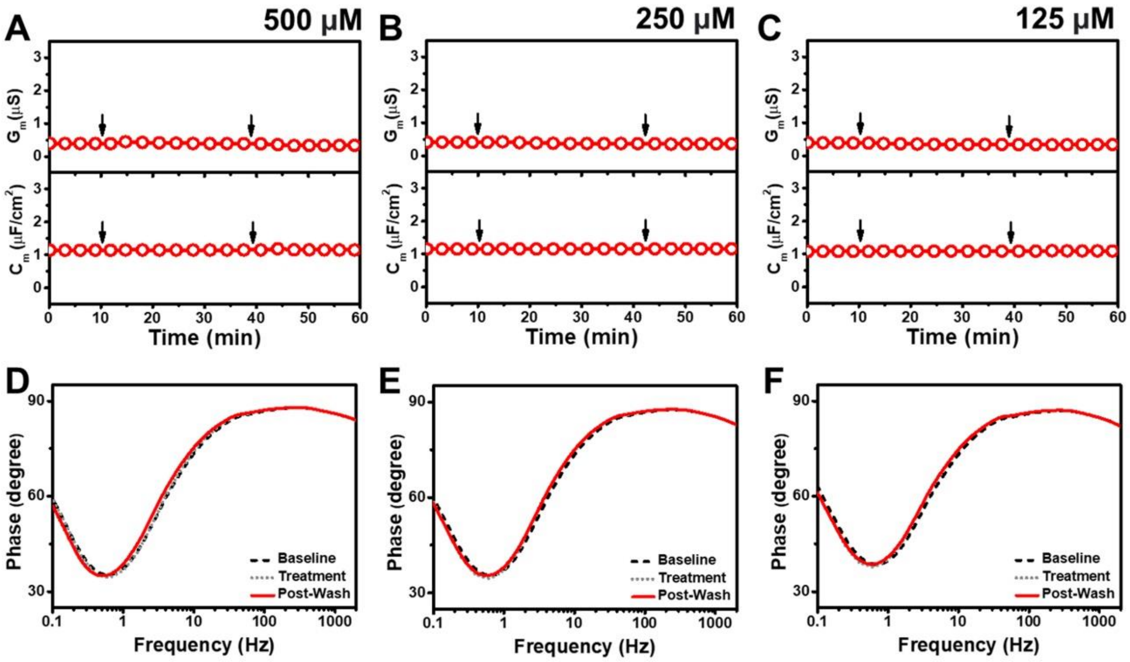

Figure S1. Time-resolved G_m and C_m shifts for low Simulsol concentrations. Data for addition of **(A)** 500 uM, **(B)** 250 μM, or **(C)** 125 μM Simulsol (first arrow) to the tBLM platform followed by subsequent buffer washing (second arrow). (**D-F)** Corresponding Bode plots.

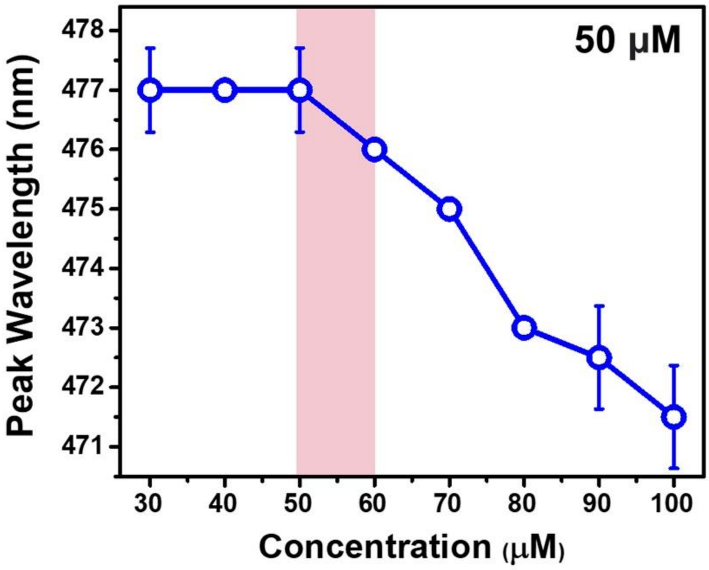

Figure S2. Critical micelle concentration (CMC) measurement of CTAB using wavelength-shift fluorescence spectroscopy. The measured peak wavelength of the fluorescent probe is plotted against CTAB concentration. The CMC value is the highest wavelength at which no peak shift occurred relative to the baseline. Each data point represents the mean and standard deviation (error bar) of *n*=4 technical replicates.

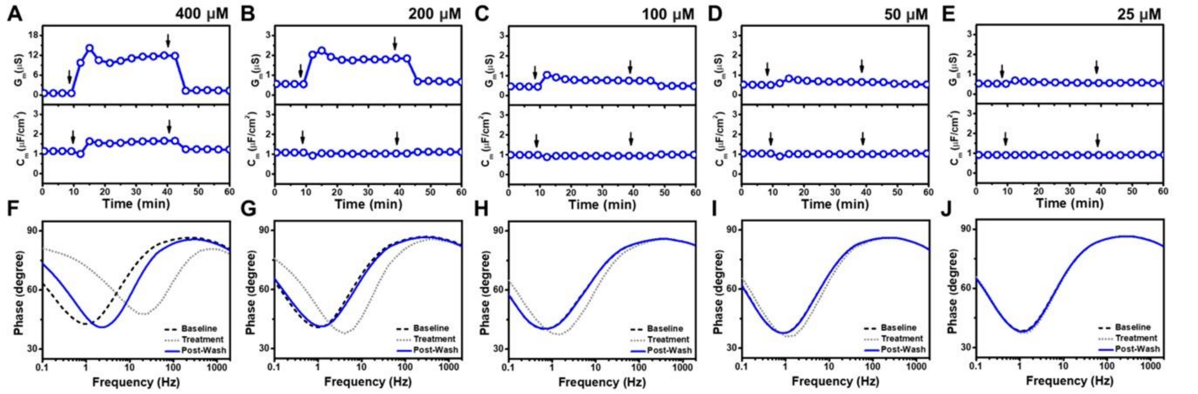

Figure S3. EIS measurements for a tBLM treated with TX-100 at equivalent concentrations to those in the CTAB experiments. Time-resolved conductance (G_m) and specific capacitance (C_m) signals following the addition of **(A)** 400 μM, **(B)** 200 μM, **(C)** 100 μM, **(D)** 50 μM, and **(E)** 25 μM TX-100. Sequential arrows indicate compound addition and buffer washing. **(F-J)** Corresponding Bode plots showing phase minima shifts vs. frequency for the tBLM baseline (Baseline), after TX-100 addition (Treatment), and after buffer rinsing (Post-Wash).

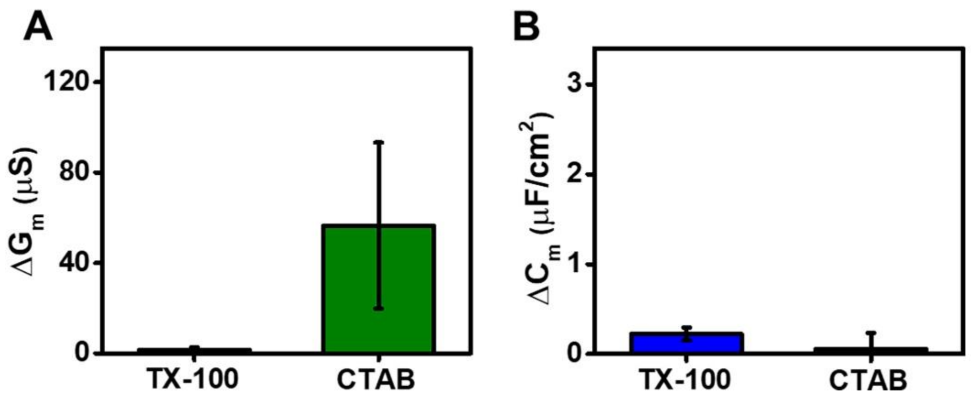

Figure S4. Experimental summary of direct comparison between 400 μM TX-100 and CTAB treatment effects. Final **(A)** G_m and **(B)** C_m shifts for the tBLM platform after treatment with 400 μM TX-100 or 400 μM CTAB followed by buffer washing (*n*=3 independent experiments). In panel (A), the differences between TX-100 vs. CTAB were statistically significant (**P*<0.05) by one-tailed, unpaired t-test. In panel (B), the differences between TX-100 vs. CTAB were not statistically significant.
